# Notes and Notices

**Published:** 1884-12

**Authors:** 


### NOTES AND NOTICES.

Removal.—Dr. Maria N. Johnson has removed from 559 North Fifteenth Street, to 1732 Green Street. Philadelphia.

Dr. C. S. Fahnstock, formerly of Indiana, and three times the President of the Indiana Institute of Homœopathy, has been recently elected to the chair of surgery in the Homœopathic Medical College of Missouri. Dr. Fahnstock is an able man, a fine operator, and will add strength to the faculty.

Laying a Corner-stone.—The corner-stone of the new building for the Hahnemann Medical College of this city was recently laid with Masonic ceremonies. In order that Homœopathy might not be altogether overlooked in the College, copies of Hahnemann's *Organon* and of The Homœopathic Physician were put in the corner-stone.

A New Journal.—The students of the Homœopathic Medical College of New York have recently started a semi-monthly journal, called *The Chironian*; its purpose is to "further, by every honorable means within its power, the interests of the College." This is certainly a laudable purpose and we wish it all success. The first number is well-gotten up.

On page 6, we are told that this College has recently introduced a new department. Its professor lectures on "the use and preparation of ointments, purgatives, emetics, poultices, etc." If this professor is expected to demonstrate the *use* of these old-fashioned methods of practice in *homœopathic* practice, he will have a hard task. As well might an Edison lecture on the preparation and use of tallow candles as a homœopath on the *use* of purgatives, etc.

Lippe's Repertory: Second Edition, revised, rewritten and much enlarged. Additions principally from Bönninghausen's Repertory—never before translated into English—which is a complete repertory to Hahnemann's works. Also many additions from *Jahr's Symptomen Codex, Lee's Cough Repertory*, etc. Volume one now *in press*. Price, $4.00; the repertory to be completed in two volumes.

As only a limited edition is to be published, those desiring this repertory will do well to send orders promptly to A. L. Chatterton Publishing Company, New York City. P. O. Box 3519.

Knowledge Regarding Cholera—Nil.—It is only by constant reiteration that knowledge is finally inculcated and public opinion affected. In this we find a justification for the frequent and discursive contributions to the subject of cholera which are at present filling the columns of the secular and medical press. The inquiring reader begins each article with fresh expectation of enlightenment. But all the knowledge of our most erudite sanitarians and eminent pathologists seems reducible to this: that cholera is communicable and portable, that it affects filthy localities and people, that rigid municipal and personal sanitation and a discriminating use of quarantine measures should be enforced.

That there is some kind of a cholera germ all believe, but what it is none know. It has been very generally agreed that this germ requires a special nidus for its development, that for the spread of cholera there must be a something which has been called "epidemic constitution." But modern sanitary reformers assert that this so-called "constitution" is only an intensity of filth. In the city of Hygeia there are no epidemic constitutions. And such is the safest practical view to take, whether it be an absolutely true one or not. * * * The cholera germ may be positively discovered any day; meanwhile Koch's bacillus theory has not, as was first hoped, thrown any flood of light upon cholera problems, and as for its prophylaxis, it is still expressed in these three phrases: *Rational quarantine, municipal cleanliness, personal hygiene.—Medical Record.*

